# Comment on Iacobescu et al. Evaluating Binary Classifiers for Cardiovascular Disease Prediction: Enhancing Early Diagnostic Capabilities. *J. Cardiovasc. Dev. Dis.* 2024, *11*, 396

**DOI:** 10.3390/jcdd13010046

**Published:** 2026-01-13

**Authors:** Mohamed Eltawil, Laura Byham-Gray, Yuane Jia, Neil Mistry, James Parrott, Suril Gohel

**Affiliations:** 1 Department of Health Informatics, School of Health Professions, Rutgers University, Newark, NJ 07107, USA; nsm69@sph.rutgers.edu (N.M.); gohelsu@shp.rutgers.edu (S.G.); 2 Department of Clinical and Preventive Nutrition Sciences, School of Health Professions, Rutgers University, Newark, NJ 07107, USA; byhamgld@shp.rutgers.edu; 3 Department of Interdisciplinary Studies, School of Health Professions, Rutgers University, Newark, NJ 07107, USA; yj340@shp.rutgers.edu (Y.J.); parrotja@shp.rutgers.edu (J.P.)

**Keywords:** machine learning, cardiovascular disease, data leakage, k-Nearest Neighbors (kNN), SMOTE-ENN, model validation, class imbalance, reproducibility, medical AI, performance evaluation

## Abstract

Machine learning is increasingly applied to cardiovascular disease prediction yet reported performance metrics often appear implausibly high due to methodological errors. Recent work has reported nearly perfect predictive accuracy (≈99%) using a k-Nearest Neighbors (kNN) model on CDC heart-disease data. Such performance greatly exceeds typical BRFSS-based benchmarks and strongly indicates data leakage. In this commentary, we replicate and re-analyze the original workflow, showing that the authors applied the SMOTE-ENN resampling method prior to the train/test split, thereby allowing synthetic data generated from the full dataset to contaminate the test set. Combined with an excessively small neighborhood parameter (k = 2), this produced misleadingly high accuracy. It is noted that (1) with SMOTE-ENN performed globally, synthetic samples appear nearly identical to test points, leading to near-perfect classification, and (2) this kNN choice is unusually small for a dataset of this scale and further amplifies leakage bias. Correcting the workflow by restricting oversampling to the training data or using undersampling restores realistic results, reducing predictive accuracy to approximately 80%, confirming the inflation caused by pre-split resampling and aligning with literature norms. This case underscores the critical importance of rigorous validation, transparent reporting, and leakage-free pipelines in medical AI. We outline practical guidelines for avoiding such pitfalls and ensuring reproducible, realistic, and clinically reliable machine-learning studies.

## 1. Introduction

Machine learning shows promise for cardiovascular risk prediction and early diagnosis of heart disease [1,2]. A recent study by Iacobescu et al. [3] evaluated several classifiers on a widely used CDC dataset and reported extraordinarily high performance, with k-Nearest Neighbors (kNN) achieving 99% accuracy and AUC ≈ 0.99. These near-perfect metrics far exceed typical results in heart disease prediction and raise serious concerns [4,5]. In this commentary, we show that data leakage likely inflated these results, as well as share concerns about the unusually small k (neighbors = 2) used in the kNN model. We share re-analysis findings that correct these issues, yielding more realistic performance, and discuss lessons for robust model evaluation in medical AI.

## 2. Unusually High Performance Raises Concerns

Near 100% accuracy in predicting cardiovascular disease is prima facie implausible and inconsistent with existing literature [4,5]. Most machine learning models for heart disease, even with advanced techniques, report far more modest accuracy and AUC values [2,3]. Thus, Iacobescu et al.’s claim that a kNN model “stood out with the highest accuracy and F1-score” at essentially 99% is an extreme outlier. The authors explicitly describe the sequence: “Following data cleaning, feature engineering, and addressing class imbalance, the next step was data transformation … [min–max] normalization … Finally, the dataset was divided into training and testing subsets (70:30)”. Absent trivial problems (e.g., an intrinsic duplication in the dataset leading to similar training and testing sets), such performance suggests a leakage or overfitting issue in the evaluation procedure as shall be further explained here.

Two red flags stand out in the study’s methodology: (1) the handling of data resampling for class imbalance, and (2) the choice of kNN with k = 2 neighbors. We discuss each below.

## 3. Data Leakage Through Improper Resampling

Data leakage occurs when there is unintended sharing of information between training and test data, leading to over-optimistic model performance. A common pitfall is performing data pre-processing (e.g., normalization, feature selection, or oversampling) before splitting the dataset, which can allow knowledge of the test set to influence the model [6].

Several concrete examples from the healthcare domain illustrate the deceptive performance caused by data leakage. In neuroimaging analysis, Yagis et al. [7] demonstrated that using a flawed validation strategy can drastically inflate results: they found that splitting MRI scans on a per-image basis (rather than by unique patients) erroneously boosted diagnostic accuracy by 30–50%, because the same patient’s images appeared in both training and testing sets. Another study showed that allowing any overlap in subjects or using feature selection with knowledge of test data inflated prediction performance significantly, whereas properly segregating data eliminated this illusion of high accuracy [8]. In clinical prediction tasks, including variables that are definitive for the diagnosis can lead to nearly perfect performance during testing—an obvious red flag.

In Iacobescu et al.’s study, the dataset (drawn from a large health survey) was heavily imbalanced (only ~8% positive for heart disease). The authors employed the SMOTE-ENN technique (Synthetic Minority Oversampling followed by Edited Nearest Neighbors) to balance classes, expanding the data from 306,939 to 472,485 records. Crucially, this resampling was performed on the entire dataset prior to the train–test split, as stated by the authors: “Following … addressing class imbalance, the next step was … the dataset was divided into training and testing subsets (70:30)” (i.e., the pipeline follows those steps: cleaning/feature-engineering → SMOTE-ENN → normalization → split). In other words, synthetic minority examples were generated using information from all data, including the eventual test set, and only afterward was the data split into training and test sets. This constitutes a textbook example of data leakage [6,9,10,11]. It means the model was effectively trained on a dataset that already contained engineered points influenced by test-set examples. As a result, the test data was no longer truly independent.

When oversampling is applied before the train/test split, some synthetic samples in the training set can be near-duplicates of test instances (or vice versa), making classification trivial. The model can “cheat” by learning from those synthetic points. This directly resonates with Iacobescu et al.’s situation—a nearly perfect kNN result likely masks a methodological flaw.

## 4. The kNN = 2 Issue and Overfitting

The grid search hyperparameter tuning selected a kNN model with k = 2 (using Euclidean distance and uniform weights). A very small neighborhood size like k = 2 (or the extreme case k = 1) often indicates a high-variance, overly complex model that can overfit the training data noise or specific patterns [12,13]. With k = 2, the classifier essentially makes decisions based on the two closest data points in the feature space [14]. In a massive dataset (over 300k samples), one would expect a somewhat larger k to be optimal for smooth generalization; a tiny k implies the model is leveraging extremely local relationships. If the training data has near-duplicate or synthetically generated points, a k = 2 classifier can exploit them to perfectly classify those training instances—but this will not translate to truly new data. In other words, the choice of k = 2 likely compounded the leakage: the model zeroed in on nearest neighbors that were often artificially similar to the query (due to SMOTE-ENN across the whole dataset), yielding a falsely high accuracy.

## 5. Results After Correcting the Methodology

We used BRFSS 2021 and the same 18 features described by Iacobescu et al. (Table 1), starting from *n* ≈ 306,939 after cleaning to reconstruct their analysis and further test alternative corrections [3]. We implemented three pipelines:

1. Reconstruction (global SMOTE-ENN + min–max, then 70/30 split)—following the exact same workflow as in [3] with the aim to replicate—as close as possible—the results established;
2. Leak-free resampling (split first; SMOTE–ENN and scalers fit only inside training folds)—limiting the application of SMOTE-ENN only to the training set, maintaining the ‘blindness’ of the model to the test set, and evaluating the model on a truly held-out 30% test set;
3. Undersampling (split first; RandomUnderSampler within folds)—using the same exact workflow in [3] while replacing the oversampling with undersampling (thus avoiding the data synthesis and leakage to the test set).

Eliminating the leakage, the overall findings in approaches (2) and (3) changed substantially from (1) (Table 1). All classifiers perform at much lower levels but are more consistent with prevailing results in the literature, and the reported gap between kNN and other algorithms closes. In fact, algorithms like Random Forest and Gradient Boosting, which are often strong performers in tabular data, achieve comparable if not better-balanced accuracy once proper validation is in place. The kNN model’s accuracy falls to a level commensurate with known benchmarks—roughly in the 80% range. The dramatic drop in kNN’s metric (around 15 points) underscores how the original evaluation was misleading. It was not that kNN is an unexpectedly high-performing model for heart disease, but rather that the evaluation leak gave it an unrealistic advantage. Once leakage is corrected, no model in our experiment approached 99% accuracy on the test set which is the expected result given the complexity of cardiovascular risk prediction.

It is important to note that class imbalance handling itself is not the culprit—indeed, techniques like SMOTE, when correctly used on training data only, can improve model learning for minority classes. The key lesson is that the train–test-split must be sacrosanct boundaries. Any operation that learns from the entire dataset (especially one creating synthetic data points) must be confined to the training portion during each fold. Iacobescu et al.’s oversight of failing to maintain the train/test split otherwise invalidated their impressive numbers.

Our replication shows that the reported 99% accuracy is not achievable under a leakage-free pipeline. This aligns with broader concerns: methodological pitfalls that inflate performance [10], with leakage identified as a root cause of the reproducibility crisis in ML-based science [11].

## 6. Other Issues Noted

In addition to the leakage, the paper used mean squared error (MSE) as the loss function for a binary neural network model—an unconventional choice for classification (where perhaps binary cross-entropy is more standard [15]). Furthermore, the terms “validation” and “testing” are used interchangeably without describing a held-out test set or nested validation, limiting reproducibility [16].

## 7. Discussion and Recommendations

This case shows how inadvertent data leakage can lead to deceptively high performance and misguided conclusions. It also highlights the responsibility of researchers to ensure evaluation rigor, especially in medical AI, when overestimating a model’s accuracy could have real clinical repercussions. Given the criticality of medical AI applications, several considerations need to be followed to draw utility from such analysis [10,17,18,19]. We summarize the following recommendations for future studies to avoid similar pitfalls:

- Split Data Early and Properly: Always separate the test set (or use cross-validation folds) before any resampling, normalization, or feature engineering steps. This ensures the model is evaluated on truly unseen data.
- Use Nested Validation for Tuning: Hyperparameter tuning (e.g., GridSearchCV) should be performed within a training fold, with an independent validation mechanism, rather than on the full dataset. This prevents “peeking” at test data during model selection.
- Apply Oversampling Only to Training Data: Techniques like SMOTE should never have knowledge of the entire dataset. Generate synthetic samples after splitting, within the training subset (and if using cross-validation, redo it for each fold). This avoids contaminating the test set with synthetic points derived from it.
- Be Wary of Extreme Metrics: Treat near-100% results with healthy skepticism. Examine whether any feature or preprocessing step could be unintentionally leaking information. Often, a deep dive will reveal either data leakage, label proxy features, or an overly simplistic dataset if performance is too good to be true.
- Cross-Check Model Complexity: If an automated search selects an unusual hyperparameter (e.g., k = 1 or 2 in kNN, very deep trees, etc.), consider if this may be overfitting. Manually inspect performance on validation vs. training sets. A small k in kNN yielding huge accuracy gains is a hint to double-check the data pipeline for leaks or anomalies. Hyperparameter choices optimized purely by an algorithm should be interpreted in the context of clinical goals—ensuring that the resulting model serves meaningful and generalizable predictions rather than just maximizing mathematical metrics.
- Report Methodology Transparently: Provide clear details on when each preprocessing step was performed relative to splitting. Ambiguity in this can hide leakage. Diagrams are helpful, but they must include these details, not just a high-level pipeline. Transparent reporting allows others to trust and reproduce the findings or catch issues if present.

The impressive results of Iacobescu et al.’s CVD prediction model were likely an artifact of evaluation errors rather than a breakthrough in classifier capability. Once corrected, the kNN model does not in fact vastly outperform more established algorithms, nor does it reach the virtually perfect accuracy originally claimed. The dataset and approach to combining risk factors remain valuable despite the methodological flaws. But the case demonstrates that rigorous validation matters in machine learning studies. Especially in healthcare applications, we must ensure our models are truly generalizing and not just “learning the test by heart.” Leakage-induced overestimation in medical AI is not a technical nuisance but a patient-safety risk—misleading clinicians and overstating model readiness for deployment. By avoiding leakage and adhering to sound evaluation practices, future research can build on these results in a reliable way, helping translate AI advances into genuine clinical utility rather than illusory performance.

## Figures and Tables

**Table 1:** Comparative accuracy results across cardiovascular prediction models.

Accuracy/ Method	Iacobescu et al. [3]	Reconstruction (1)	Proper Sampling (2)	Undersampling (3)
Random Forest	0.94	0.95	0.84	0.75
Log. Reg.	0.88	0.85	0.68	0.76
kNN	0.99	0.92	0.77	0.66
Grad. Boosting	0.95	0.95	0.87	0.74

## Data Availability

The dataset used in this study is publicly available from the CDC Behavioral Risk Factor Surveillance System (BRFSS). The Python Jupyter notebook used for building the pipeline (data preprocessing, resampling, model training, and evaluation) is publicly available at https://github.com/eltawilm/CVD_Classification_on_BRFSS (accessed on 18 September 2025). The code includes the pipeline scripts replicating Iacobescu et al.’s workflow and our corrected (leak-free) re-evaluation. The BRFSS 2021 dataset is publicly available from the CDC (downloadable from https://www.cdc.gov/brfss/annual_data/2021/files/LLCP2021XPT.zip, accessed on 18 September 2025) and not redistributed here. The analysis code is available upon request from the corresponding author.

## References

[1] Dey D., Slomka P.J., Leeson P., Comaniciu D., Shrestha S., Sengupta P.P., Marwick T.H. (2019). Artificial Intelligence in Cardiovascular Imaging. J. Am. Coll. Cardiol..

[2] Krittanawong C., Virk H.U.H., Bangalore S., Wang Z., Johnson K.W., Pinotti R., Zhang H., Kaplin S., Narasimhan B., Kitai T. (2020). Machine learning prediction in cardiovascular diseases: A meta-analysis. Sci. Rep..

[3] Iacobescu P., Marina V., Anghel C., Anghele A.-D. (2024). Evaluating Binary Classifiers for Cardiovascular Disease Prediction: Enhancing Early Diagnostic Capabilities. J. Cardiovasc. Dev. Dis..

[4] Van Calster B., Nieboer D., Vergouwe Y., De Cock B., Pencina M.J., Steyerberg E.W. (2016). A calibration hierarchy for risk models was defined: From utopia to empirical data. J. Clin. Epidemiol..

[5] Ioannidis J.P.A. (2005). Why Most Published Research Findings Are False. PLoS Med..

[6] Alturayeif N., Hassine J. (2025). Data leakage detection in machine learning code: Transfer learning, active learning, or low-shot prompting?. PeerJ Comput. Sci..

[7] Yagis E., Atnafu S.W., de Herrera A.G.S., Marzi C., Scheda R., Giannelli M., Tessa C., Citi L., Diciotti S. (2021). Effect of data leakage in brain MRI classification using 2D convolutional neural networks. Sci. Rep..

[8] Rosenblatt M., Tejavibulya L., Jiang R., Noble S., Scheinost D. (2024). Data leakage inflates prediction performance in connectome-based machine learning models. Nat. Commun..

[9] Demircioğlu A. (2024). Applying oversampling before cross-validation will lead to high bias in radiomics. Sci. Rep..

[10] Maleki F., Ovens K., Gupta R., Reinhold C., Spatz A., Forghani R. (2023). Generalizability of Machine Learning Models: Quantitative Evaluation of Three Methodological Pitfalls. Radiol. Artif. Intell..

[11] Kapoor S., Narayanan A. (2023). Leakage and the reproducibility crisis in machine-learning-based science. Patterns.

[12] Batista G.E.A.P.A., Prati R.C., Monard M.C. (2004). A study of the behavior of several methods for balancing machine learning training data. ACM SIGKDD Explor. Newsl..

[13] Hastie T., Tibshirani R., Friedman J.H. (2009). The Elements of Statistical Learning: Data Mining, Inference, and Prediction.

[14] Cover T., Hart P. (1967). Nearest neighbor pattern classification. IEEE Trans. Inf. Theory.

[15] Terven J., Cordova-Esparza D.-M., Romero-González J.-A., Ramírez-Pedraza A., Chávez-Urbiola E.A. (2025). A comprehensive survey of loss functions and metrics in deep learning. Artif. Intell. Rev..

[16] Krstajic D., Buturovic L.J., Leahy D.E., Thomas S. (2014). Cross-validation pitfalls when selecting and assessing regression and classification models. J. Cheminform..

[17] Sasse L., Nicolaisen-Sobesky E., Dukart J., Eickhoff S.B., Götz M., Hamdan S., Komeyer V., Kulkarni A., Lahnakoski J.M., Love B.C. (2025). Overview of leakage scenarios in supervised machine learning. J. Big Data.

[18] Sasse L., Nicolaisen-Sobesky E., Dukart J., Eickhoff S.B., Götz M., Hamdan S., Komeyer V., Kulkarni A., Lahnakoski J., Love B.C. (2024). On Leakage in Machine Learning Pipelines. arXiv.

[19] Lones M.A. (2021). How to avoid machine learning pitfalls: A guide for academic researchers. arXiv.

